# Vital Role of *Glutamate Dehydrogenase* Gene in Ammonia Detoxification and the Association Between its SNPs and Ammonia Tolerance in *Sinonovacula constricta*

**DOI:** 10.3389/fphys.2021.664804

**Published:** 2021-05-05

**Authors:** Gaigai Sun, Yinghui Dong, Changsen Sun, Hanhan Yao, Zhihua Lin

**Affiliations:** ^1^College of Fisheries, Henan Normal University, Xinxiang, China; ^2^Ninghai Institute of Mariculture Breeding and Seed Industry, Zhejiang Wanli University, Ninghai, China; ^3^Key Laboratory of Aquatic Germplasm Resources of Zhejiang, College of Biological and Environmental Sciences, Zhejiang Wanli University, Ningbo, China

**Keywords:** *Sinonovacula constricta*, GDH, ammonia stress, SNP, immunohistochemistry, RNAi

## Abstract

Increasing evidence has revealed accumulated ammonia will cause adverse effects on the growth, reproduction, and survival of aquatic animals. As a marine benthic mollusk, the razor clam *Sinonovacula constricta* shows better growth and survival under high ammonia nitrogen environment. However, little is known about its adaptation mechanisms to high ammonia stress in an integrated mariculture system. In this study, we analyzed the association between the polymorphism of *glutamate dehydrogenase* gene (*GDH*), a key gene involved in ammonia nitrogen detoxification, and ammonia tolerance. The results showed that 26 and 22 single-nucleotide polymorphisms (SNPs) of *GDH* in *S. constricta* (denoted as *Sc-GDH*) were identified from two geographical populations, respectively. Among them, two SNPs (c.323T > C and c.620C > T) exhibited a significant and strong association with ammonia tolerance, suggesting that *Sc-GDH* gene could serve as a potential genetic marker for molecular marker–assisted selection to increase survival rate and production of *S. constricta*. To observe the histological morphology and explore the histocellular localization of Sc-GDH, by paraffin section and hematoxylin–eosin staining, the gills were divided into gill filament (contains columnar and flattened cells) and gill cilia, whereas hepatopancreas was made up of individual hepatocytes. The results of immunohistochemistry indicated that the columnar cells of gill filaments and the endothelial cells of hepatocytes were the major sites for Sc-GDH secretion. Under ammonia stress (180 mg/L), the expression levels of *Sc-GDH* were extremely significantly downregulated at 24, 48, 72, and 96 h (*P* < 0.01) after RNA interference. Thus, we can speculate that *Sc-GDH* gene may play an important role in the defense process against ammonia stress. Overall, these findings laid a foundation for further research on the adaptive mechanisms to ammonia–nitrogen tolerance for *S. constricta*.

## Introduction

In practice, some improper management methods such as high-density stocking and overfeeding lead to gradual ammonia nitrogen accumulation in rearing water, which are extremely toxic to the health of aquatic organisms. Ammonia is usually present in ionized (${\text{NH}}_{4}^{+}$) and unionized (NH_3_) states in water, and NH_3_ diffuses easily across membrane and into hemolymph of aquatic animals (Randall and Tsui, 2002). A number of studies have demonstrated that ammonia exposure resulted in histopathologic changes in gill and liver of *Oreochromis niloticus* (Benli et al., 2008), *Cyprinus carpio* (Peyghan and Takamy, 2002), and *Portunus pelagicus* (Romano and Zeng, 2006). Similarly, the gills of sliver catfish exposed to ammonia levels above *LC*_50_ (96 h) resulted in edema and fusion of the secondary lamellae (Miron et al., 2008). Additionally, the ammonia exposure induces oxidative stress and then further causes endoplasmic reticulum stress and apoptosis in the hepatopancreas of *Litopenaeus vannamei* (Liang et al., 2016). A similar pattern was observed in *Ruditapes philippinarum* showing that ammonia can destroy the stability of lysosomal membrane and cause cell apoptosis of the gills (Cong et al., 2017). However, the histocellular localization of genes associated with ammonia–nitrogen excretion in the gills and liver has been rarely reported. It should also be mentioned that *Opsanus beta* and *Porichthys notatus* exhibited remarkably different levels of the branchial expression of nitrogen transporters, both with regard to urea transporter proteins and Rhesus glycoprotein proteins, in accord with their disparate approaches to nitrogen metabolism (Bucking et al., 2013). Similarly, the changes of protein expression in the Na^+^/K^+^-ATPase and Rhesus glycoprotein C like of sea lamprey (*Petromyzon marinus*) gill during metamorphosis suggested that the methods of excreting ammonia were fundamentally changed (Sunga et al., 2020). Another study on immunofluorescence indicated that ammonium transporters might participate in ammonia excretion instead of ammonia absorption and assimilation in *Tridacna squamosa* (Boo et al., 2018). However, to date, little research focuses on the action site of genes related to detoxification metabolism of benthic mollusks exposed to high ammonia.

The razor clam *Sinonovacula constricta* is an economically important bivalve species along the coast of west Pacific Ocean, which has become one of the most popular seafood because of its delicious taste. In China, the clam yield is mainly obtained by polyculture with shrimps or crabs in seawater pond (Xie et al., 2011; Li et al., 2016). *S. constricta*, as a benthic bivalve, often lives in the sand mud from the surface to a depth of 40–50 cm in a comprehensive aquaculture system, so it always faces more severe ammonia nitrogen than other species because of the deposition and decay of surplus food and dead organisms in the sediment. However, it is still unclear about its underlying molecular mechanism to ammonia nitrogen detoxification. Long-term exposure to ammonia eventually leads to increased susceptibility of adult bivalves and embryos to contaminant stress or reduced reproductive capacity (Keppler, 2007), although mollusks have a high tolerance to pollutants (Saha et al., 2000). By comparing several marine bivalves, it has been found that the benthic species, such as *R. philippinarum* (Cong et al., 2019) and *Corbicula fluminea* (Zhang et al., 2019), had higher ammonia nitrogen tolerance than adhesive ones, such as oyster (Epifanio and Srna, 1975) and scallop (Widman et al., 2008). In this regard, different mollusks may have specific adaptive strategies to high concentration of ammonia.

It is commonly known that *glutamate dehydrogenase* (*GDH*) is a mitochondrial enzyme and widely exists in plants (Kanamori et al., 1972), animals (Spanaki and Plaitakis, 2012), and microorganisms (Kanamori et al., 1987). It can catalyze the reversible reaction α-ketoglutarate + NH_3_ + NADH ⇆ glutamate + NAD^+^ and is allosterically regulated by leucine, pyridine, adenine, and guanine nucleotides (Smith et al., 2001). In general, GDH maintains the low concentration of ammonia in the body by converting excess ammonia into glutamate when the concentration of ammonia nitrogen is higher than normal level (Cooper, 2012). Researches on the *GDH* gene in aquatic animals mainly focus on fish and crustaceans, such as *Monopterus albus* (Tng et al., 2010) and *Penaeus monodon* (Yang et al., 2015). By contrary, *GDH* gene has been rarely studied in mollusks. In our previous study, we found that the expression of *Sc-GDH* was significantly increased under acute ammonia stress (*P* < 0.05). Further, the results of quantitative real-time polymerase chain reaction (qRT-PCR) showed that the expression of *Sc-GDH* was higher in the gill and hepatopancreas than other tissues (*P* < 0.05) (Zhang et al., 2020). Therefore, we speculated that *Sc-GDH* gene is involved in ammonia tolerance by influencing the active site. To investigate this hypothesis, in this study, we identified the polymorphisms of *Sc-GDH* gene and analyzed its association with ammonia tolerance. Then, the histological structure of gills and hepatopancreas was observed, in order to further explore the histocellular localization of Sc-GDH protein by immunohistochemistry method. Furthermore, we performed an RNA interference (RNAi) experiment to analyze the expression levels of *Sc-GDH*. These works would help us understand ammonia adaptation mechanism and develop a viable source for the breeding of new ammonia-tolerant variety in *S. constricta*.

## Materials and Methods

### Ethics Statement

The razor clams *S. constricta* used in this work were collected from the genetic breeding research center of Zhejiang Wanli University, China. All experimental procedures were approved by the Institutional Animal Care and Use Committee of Zhejiang Wanli University, China.

### Ammonia Challenge and Sample Collection

Two geographical populations of *S. constricta* with an average shell length of 63.26 ± 1.73 mm from the coastal areas of Ninghai, Zhejiang province (ZJ), and Changle, Fujian province (FJ), in China were collected and raised in the genetic breeding research center of Zhejiang Wanli University. All these clams were acclimatized in seawater with a salinity level of 22 at 22 ± 0.5°C with aeration and were fed with *Chaetoceros muelleri* in the morning and evening.

For ammonia challenge experiments, 1,200 individuals from each population were divided into five groups (240 clams per group): the control group (CG) and four ammonia stress groups (AG). The CG tank was filled with 500 L of natural seawater, while the four AG tanks were filled with 500 L of seawater with a high concentration of ammonia (180 mg/L), according to the 96-h LC50 values (Zhang et al., 2020). The ammonia concentration in the AG was adjusted by adding NH_4_Cl (Sangon, Shanghai, China) solution to the seawater, and the pH value was calibrated to 7.87 ± 0.23 by 10% NaOH solution. All the clams were monitored to eliminate the dead individuals every 2 h. The stress exposure lasted for 120 h. The surviving clams throughout the challenge experiment in the AGs of each population were classified as tolerant group (TG). Afterward, the gills of selected samples were dissected and immediately frozen in liquid nitrogen for RNA extraction. Moreover, the gills and hepatopancreas of six surviving individuals from the AG and CG from ZJ population were collected for paraffin section and immunohistochemistry test.

### Primers and PCR Amplification

Total RNAs were extracted from gills using Trizol reagent (Omega, United States) according to the manufacturer’s instructions. RNA purity was measured using the NanoDrop 2000 spectrophotometers (Thermo Scientific, United States). RNA degradation and contamination were monitored on agarose electrophoresis. First-stand cDNA was synthesized from total RNA using PrimeScript RT reagent kit with gDNA Eraser (Takara, Japan). The thermal cycles were conducted in a PCR instrument (Bio-Rad, United States). Gene-specific primers were designed based on the sequence of *Sc-GDH* gene (GenBank accession no. MK451702) (Table 1). The amplifications were performed in a total volume of 25 μL, which contained 12.5 μL of Taq PCR Master Mix (Sangon, Shanghai, China), 1 μL of each primer (10 μM), 9.5 μL of PCR-grade water, and 1 μL of cDNA template. The PCR was carried out with the following conditions: 35 cycles of 95°C for 30 s, 58°C for 30 s, and 72°C for 1 min and a final extension of 72°C for 10 min. After amplification, the PCR products were detected by electrophoresis on 1% agarose gels. Specific PCR products were purified and sequenced.

**TABLE 1:** Primers and sequences of the experiments.

Primer	Sequence (5′–3′)	Comment
siRNA-*GDH*-F1	GCUGGUGGUGUGACUGUAUTT	RNAi
siRNA-*GDH*-R1	AUACAGUCACACCACCAGCTT	
NC-F	UUCUCCGAACGUGUCACGUTT	RNAi
NC-R	ACGUGACACGUUCGGAGAATT	
*Sc-GDH*-F2	ACATCCTGCGTATGATCAAGCC	qRT-PCR of RNAi
*Sc-GDH*-R2	CATTCACGTCCAAACTGTATCGG	
*RS*9-F	TGAAGTCTGGCGTGTCAAGT	qRT-PCR of the reference gene
*RS*9-R	CGTCTCAAAAGGGCATTACC	
*Sc-GDH*-F3	TCGATACATCCGATTTTGG	SNP
*Sc-GDH*-R3	ATTTGAAGGTTAGACGCCC	

### Association Analysis Between the Single-Nucleotide Polymorphisms of *Sc-GDH* and Ammonia Tolerance

In the above ammonia challenge experiment, 96 and 132 survived individuals in FJ and ZJ population were regarded as the TGs, respectively. At the same time, 100 and 121 individuals in FJ and ZJ population were selected as CGs, respectively. Then, the total RNAs of all samples from the TGs and CGs were extracted, synthesized into cDNA, performed PCR amplification, and sequenced. The nucleotide sequences of the *Sc-GDH* from different individual clams were aligned using Mutation Surveyor. To detect the single-nucleotide polymorphisms (SNPs), *χ*^2^ of SPSS 22 software was employed to determine statistical significance. The observed heterozygosity (*Ho*), expected heterozygosity (*He*), effective allele (*Ne*), and polymorphism information content (*PIC*) of these SNPs were calculated by Popgen32. The Hardy–Weinberg equilibrium and associated loci linkage disequilibrium (LD) of these SNPs were analyzed by SHEsis online^1^ (Shi and He, 2006; Li et al., 2009).

### Paraffin Section, Hematoxylin–Eosin Stain, and Immunofluorescence Staining

To test the cellular localization of Sc-GDH as well as the histological morphology in gills and hepatopancreas, the conventional paraffin sections, hematoxylin–eosin (H&E) staining, and immunofluorescence staining were performed. The tissues were fixed in 4% paraformaldehyde at 4°C for 24 h and then dehydrated with graded alcohol, transparent, infiltration paraffin, and paraffin-embedded. Paraffin sections (4 μm) were deparaffinized first and stained with hematoxylin (8 min) and eosin (1 min), respectively, and then photographed under an optical microscope (Nikon Eclipse 80i, Japan). In addition, paraffin sections were deparaffinized prior to immersion in 10 mM sodium citrate buffer for 20 min for antigen retrieval at a subboiling temperature (96°C). Sections were then blocked for 1 h in blocking solution (5% albumin from bovine serum) at room temperature, followed by primary antibody (antibody rabbit anti-GDH, produced privately by HuaBio, Zhejiang, 1:200) incubation (overnight, 4°C). Optimum primary antibody dilutions were predetermined by known positive control tissues. A known positive control section was included in each step to ensure proper staining. Primary antibody was detected by secondary antibodies Alexa Flour 488 donkey anti–rabbit immunoglobulin G (Invitrogen, A21206) and diluted at 1:250. Nuclei were stained with DAPI (Beyotime, Shanghai, China). The cells were observed and photographed under a fluorescence microscope (Nikon Eclipse 80i, Japan).

### RNA Interference

Three hundred healthy adult clams selected from the genetic breeding research center of Zhejiang Wanli University (the average shell length was 62.43 ± 1.45 mm) were divided into three groups; the adductor muscles of all individuals of each group were injected with 5,000 ng diluted small interfering RNA (siRNA) of *Sc-GDH* (siRNA-*GDH*, Sangon, Shanghai, China), 5,000 ng diluted siRNA-negative control (NC), or 5,000 ng DEPC treated water (DEPC-W, blank control) and then were continuously cultured for 120-h exposure to 180 mg/L ammonia nitrogen seawater in three 500-L tanks (the management measures during the period are the same as above). The hepatopancreas tissues from six clams per treatment group were removed at 0, 24, 48, 72, 96, and 120 h postinjection.

For each sample, triplicate qRT-PCR reactions with iTaq^TM^ universal SYBR^®^ Green Supermix (Bio-Rad, CA, United States) following the manufacturer’s instructions were performed on Roche LightCycler^®^ 480 System (Roche Diagnostics, Basel, Switzerland). Each containing 2 μg of total RNA as a template was performed with cycling conditions: 95°C for 10 min, 40 cycles of denaturation at 95°C for 10 s, annealing at 60°C for 1 min, and a final extension at 72°C for 10 s. The relative expression levels of *Sc-GDH* were calculated by the 2^–Δ^ ^Δ^ ^*Ct*^ method. The ribosomal protein S9 (*RS*9) gene was used as an internal reference gene (Zhao et al., 2018). All PCR primers used in this study are listed in Table 1.

## Results

### Association Analysis Between SNPs of *Sc-GDH* and Ammonia Tolerance

**FIGURE 1:**
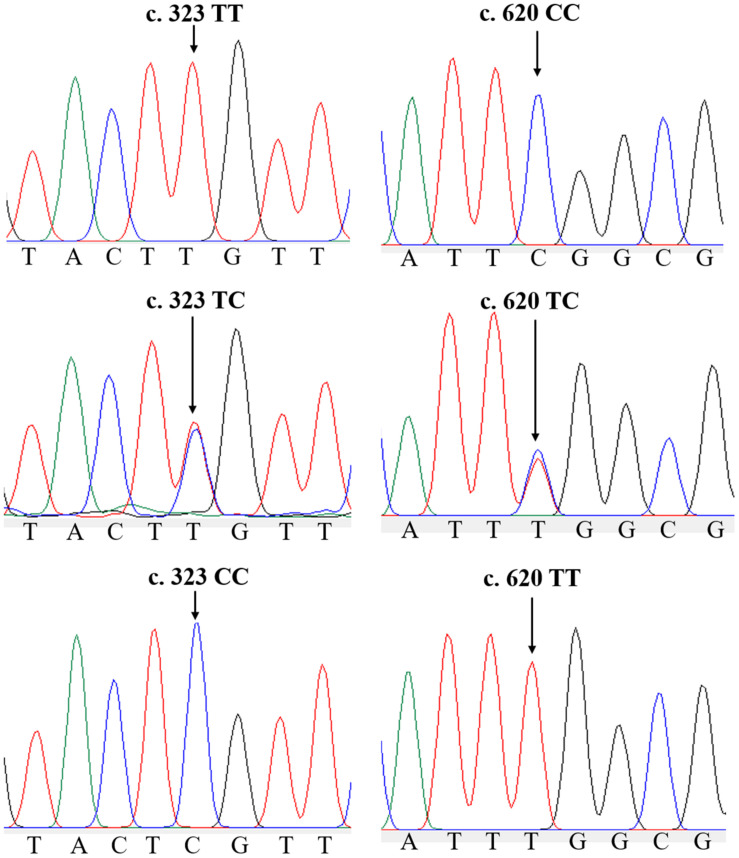
Two single-nucleotide polymorphisms (SNPs) of *Sc-GDH* gene. Peaks of normal and two mutated forms of c.323T > C and c.620C > T locus in *Sc-GDH*.

In the ammonia challenge experiment, the first dead clam was observed in ZJ population at 24 h after ammonia stress, and the mortality rate was steadily increased and reached its apex level at 88–90 h. There were no mortalities found in the CG during the experiment. In this study, a total of 26 and 22 SNPs were detected by sequence comparisons in ZJ and FJ population, respectively (Supplementary Table 1). Each SNP was named after the rank-number of bases from the first base of mRNA sequence of *Sc-GDH* to the mutation locus for convenience. All loci of the two populations were synonymous mutations. The sample size, percent genotypes, and variation type of SNPs with significant differences of *Sc-GDH* in each population are shown in Table 2. The c.323T > C and c.620C > T were significantly different in genotype frequency between AG and TG in each population (*P* < 0.05) (Table 2), speculating that they are strongly associated with ammonia tolerance. The sequencing peak map showed that there were two highly consistent peaks at the mutation locus, which makes the c.323T > C and c.620C > T changes from homozygous to heterozygous (Figure 1).

**TABLE 2:** Genotype, gene frequency, and variation type of SNPs in *Sc-GDH*.

Locus/variation type	Genotype	ZJ			FJ		
		TG/CG		*χ* ^2^/*P* value	TG/CG		*χ* ^2^/*P* value
		Number	Genotype frequency		Number	Genotype frequency	
c.269C > T/transition	CC	51/39	38.64/32.23	2.833/0.243	76/45	79.16/45	26.993/0.000
	CT	56/56	42.42/46.28		17/38	17.71/38	
	TT	25/26	18.94/21.49		3/17	3.13/17	
**c.323T** > **C**/transition	TT	46/38	34.85/31.41	**22.965/0.000**	74/39	77.08/39	**30.849/0.000**
	TC	57/25	43.18/20.66		18/44	18.75/44	
	CC	29/58	21.97/47.93		4/17	4.17/17	
c.401G > A/transition	GG	71/71	53.79/58.67	3.65/0.161	89/60	92.71/60	31.196/0.000
	GA	54/38	40.91/31.41		6/34	6.25/34	
	AA	7/12	5.30/9.92		1/6	1.04/6	
**c.620C** > **T**/transition	CC	53/27	40.15/22.31	**11.983/0.003**	20/24	20.83/24	**6.244/0.044**
	CT	57/57	43.18/47.11		30/45	31.25/45	
	TT	22/37	16.67/30.58		46/31	47.92/31	
c.677T > C/transition	TT	39/18	29.54/14.88	8.114/0.017	12/12	12.50/12	0.096/0.953
	TC	55/58	41.67/47.93		41/41	42.71/41	
	CC	38/45	28.79/37.19		43/47	44.79/47	
c.872C > T/transition	CC	106/76	80.30/62.81	10.125/0.006	74/70	77.08/70	4.032/0.133
	CT	23/42	17.43/34.71		19/20	19.79/20	
	TT	3/3	2.27/2.48		3/10	3.13/10	
c.1181T > G/transversion	TT	73/40	55.30/33.06	12.843/0.002	27/31	28.13/31	0.457/0.796
	TG	37/49	28.03/40.49		39/36	40.62/36	
	GG	22/32	16.67/26.45		30/33	31.25/33	
c.1247C > T/transition	CC	35/13	26.52/10.74	10.582/0.005	16/11	16.67/11	1.337/0.512
	CT	71/29	53.79/23.97		32/35	33.33/35	
	TT	26/79	19.69/65.29		48/54	50/54	
c.1373A > C/transversion	AA	68/31	51.51/25.62	20.448/0.000	44/34	45.83/34	4.573/0.102
	AC	46/54	34.85/44.63		36/38	37.50/38	
	CC	18/36	13.64/29.75		16/28	16.67/28	

*The loci in bold black were those showing significant differences between the two populations.*

Of the 26 SNPs in ZJ population, 23 were transversions, whereas 20 transversions were found in 22 SNPs of FJ population (Supplementary Table 1). In addition, the *Ho*, *He*, *Ne*, and *PIC* values of these SNPs in two populations are shown in Supplementary Table 2. The c.323T > C represented a moderately polymorphic in ZJ population, whereas the c.620C > T indicated a moderately polymorphic in both two populations (Table 3). Further, the LD analysis revealed the linkage relationship (*D*’ > 0.75) between the c.323T > C and c.620C > T in both populations.

**TABLE 3:** Population genetic parameters of variable loci in *Sc-GDH*.

Population	Locus	TG				CG			
		*Ho*	*He*	*Ne*	*PIC*	*Ho*	*He*	*Ne*	*PIC*
ZJ	c.269C > T	0.424	0.482	0.481	0.365	0.463	0.496	0.494	0.373
	c.323T > C	0.432	0.494	0.492	0.371	0.207	0.488	0.486	0.369
	c.401G > A	0.409	0.384	0.383	0.311	0.314	0.383	0.381	0.311
	c.620C > T	0.432	0.474	0.472	0.360	0.471	0.499	0.497	0.373
	c.677T > C	0.417	0.502	0.500	0.375	0.479	0.477	0.475	0.363
	c.872C > T	0.174	0.196	0.196	0.177	0.347	0.319	0.318	0.269
	c.1181T > G	0.280	0.427	0.425	0.336	0.405	0.500	0.498	0.374
	c.1247C > T	0.538	0.500	0.498	0.374	0.231	0.348	0.347	0.284
	c.1373A > C	0.349	0.430	0.428	0.336	0.446	0.501	0.499	0.375
FJ	c.269C > T	0.788	0.211	1.267	0.189	0.537	0.461	1.855	0.355
	c.323T > C	0.765	0.234	1.306	0.206	0.522	0.476	1.908	0.363
	c.401G > A	0.920	0.080	1.087	0.074	0.644	0.354	1.549	0.292
	c.620C > T	0.534	0.463	1.863	0.356	0.450	0.498	1.990	0.374
	c.677T > C	0.550	0.448	1.811	0.348	0.560	0.439	1.782	0.343
	c.872C > T	0.772	0.227	1.679	0.201	0.678	0.320	1.471	0.269
	c.1181T > G	0.505	0.492	1.969	0.371	0.498	0.450	1.999	0.375
	c.1247C > T	0.553	0.444	1.800	0.344	0.590	0.408	1.688	0.325
	c.1373A > C	0.540	0.458	1.843	0.353	0.499	0.498	1.993	0.374

### Histological Morphology and Histocellular Localization of Sc-GDH in Gills and Hepatopancreas

**FIGURE 2:**
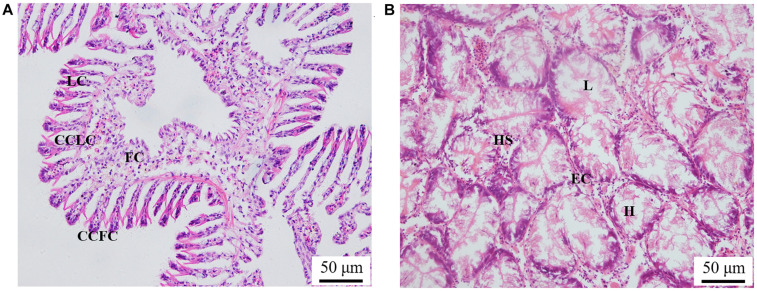
Paraffin section observation of gills in panel **(A)** and hepatopancreas in panel **(B)** in the *Sinonovacula constricta*. LC, lateral cilia; CCLC, columnar cells of lateral cilia; FC, flat cells; CCFC, columnar cells of frontal cilia; L, lumen; HS, hepatic sinusoid; EC, endothelial cells; H, hepatocytes. Scale bars were 50 μm.

**FIGURE 3:**
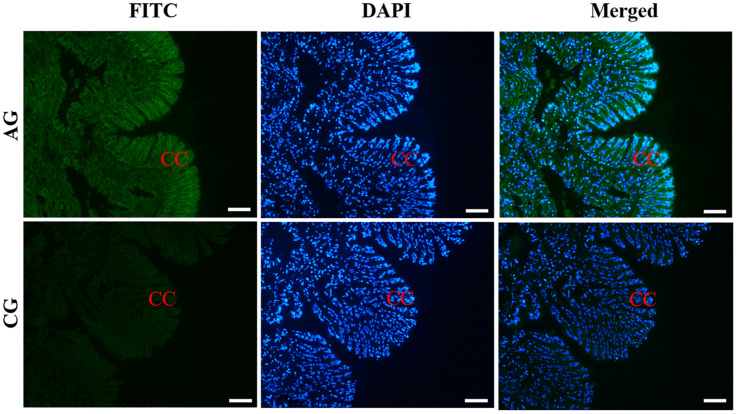
Immunofluorescence of Sc-GDH in the gills of *S. constricta*. Sc-GDH protein was stained with the anti-GDH antibody in green, and nuclei were stained with DAPI in blue. The merged images were co-localized with Sc-GDH protein (green) and DAPI (blue) in the columnar cells of lateral cilia of gills. CC, columnar cells. Scale bars were 50 μm.

The histological observation of clam gills and hepatopancreas by paraffin section and H&E staining showed that nuclei were stained with hematoxylin (blue–purple), and the cytoplasm and components of the extracellular matrix were stained with eosin (red) (Figure 2). Gills were made up of cilia and filaments composed of a single layer of epithelial cells and their surrounding blood lumen. Among them, the epithelium of gill filaments was flat cells in about half of the area near the gill lumen; the remaining epithelial cells could be subdivided into columnar cells of frontal and lateral cilia according to the position of cilia (Figure 2A). Then, we proceeded to examine the expression and histocellular localization of Sc-GDH in the gills from *S. constricta*. The fluorescein isothiocyanate (FITC)–labeled antibody (antibody mouse anti-rabbit) selected in this study can specifically label Sc-GDH protein in tissues and display green fluorescence. Compared with the FITC staining (green) in the CG, the columnar cells in lateral cilia of gill in the AG showed a strong and positive signal (Figure 3).

**FIGURE 4:**
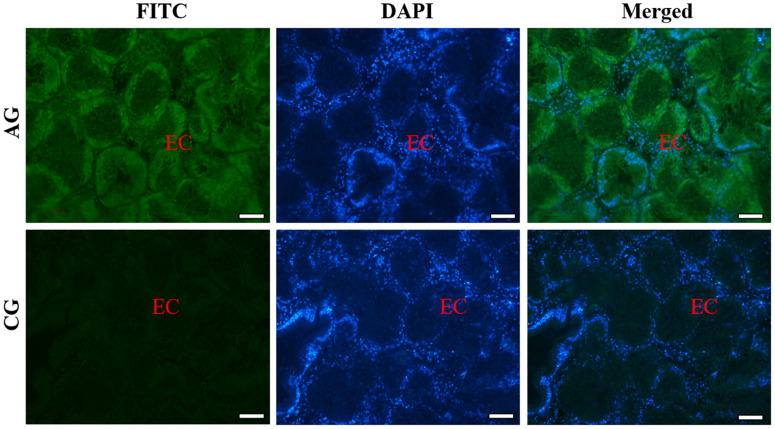
Immunofluorescence of Sc-GDH in the hepatopancreas of *S. constricta*. Sc-GDH protein was stained with the anti-GDH antibody in green, and nuclei were stained with DAPI in blue. The merged images were co-localized with Sc-GDH protein (green) and DAPI (blue) in the endothelial cells of hepatic sinusoid. EC, endothelial cells. Scale bars were 50 μm.

Similarly, the histological structure of hepatopancreas was observed, and histocellular localization of Sc-GDH was performed. Hepatopancreas was made up of individual hepatocytes, which was irregularly elliptical sphere in shape, and hepatic sinusoid was formed between each hepatocyte cell. The wall of hepatic sinusoid was made up of endothelial cells, which enhances the permeability of hepatic sinusoid, and was conducive to the material exchange between hepatocyte cell and blood (Figure 2B). In addition, the different size of the lumens in the hepatocyte cells also increases the possibility of the material exchange between hepatocyte cell and blood (Figure 2B). On this basis, Sc-GDH was detected in endothelial cells of hepatic sinusoid in AG and showed positive signal, whereas CG exhibited extremely low expression of Sc-GDH (Figure 4). The combination diagram showed that the green and the blue signal did not coincide, indicating that Sc-GDH was not expressed in the nucleus (Figures 3, 4).

### Expression of *Sc-GDH* After RNAi Silencing

**FIGURE 5:**
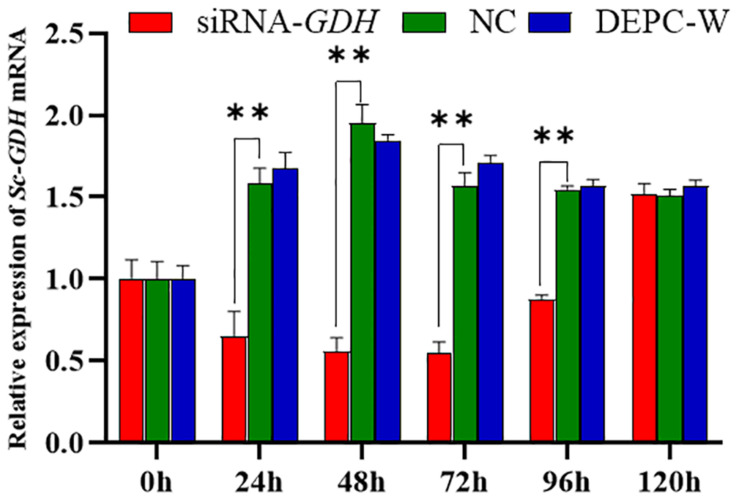
Relative expression of *Sc-GDH* genes in the hepatopancreas after RNA interference. Vertical bars represented the mean ± SD (*n* = 4). ^∗∗^Extremely significant difference between siRNA-*GDH* and NC (*P* < 0.01).

RNA interference technology was used further investigate *Sc-GDH* function in the hepatopancreas. The results showed that a variation trend of the mRNA expression level of *Sc-GDH* was roughly the same in NC and DEPC-W. In addition, the mRNA expression level of *Sc-GDH* in siRNA-*GDH* was significantly lower than that in NC at 24, 48, 72, and 96 h (*P* < 0.01), which correspondingly downregulated to approximately 58.83, 71.41, 65.25, and 43.30% (Figure 5). However, there were no significant differences at 96–120 h.

## Discussion

Up to now, researches on the *GDH* gene in aquatic animals mainly focus on fish and crustaceans, such as *Oncorhynchus mykiss* (Wright et al., 2007), *Periophthalmodon schlosseri*, *Boleophthalmus boddaerti* (Ip et al., 2005), *Clarias gariepinus* (Wee et al., 2007), *L. vannamei* (Qiu et al., 2018), and *Macrobrachium rosenbergii* (Dong et al., 2020). However, the related studies on mollusks are very scarce. In this study, we inferred that *Sc-GDH* gene may be related with the antiammonia response of clams, and selected it as a candidate gene to detect the SNPs associated with ammonia tolerance traits in *S. constricta*. Furthermore, we explored the cellular localization of Sc-GDH in gill and hepatopancreas. In addition, to investigate the functions, the expression responses of *Sc-GDH* were determined under ammonia stress. To our knowledge, these studies were first carried out in *S. constricta*.

It has been proposed that SNP, a molecular genetic marker of the third generation, has a good application prospect in population genetic analysis of aquatic animal, molecular marker–assisted selection (MAS) and biological evolution (Wenne, 2017). Increasing evidence has shown that some SNPs are associated with growth, disease resistance, and other economic traits of clams (He et al., 2012; Siva et al., 2012, 2016; Nie et al., 2015; Niu et al., 2015; Yao et al., 2020; Zhao et al., 2020). For instance, a synonymous mutation SNP c.852A > G of insulin-like growth factor 2 mRNA-binding protein gene in *Patinopecten yessoensis* showed a significant association with the growth traits (Ning et al., 2018), while two SNPs HbIIA-E2-146 and HbIIB-E2-23 of the hemoglobin gene (*Hb*) in *Tegillarca granosa* were associated with the *Vibrio parahaemolyticus* resistance (Bao et al., 2013). The +2248T/C and +2365T/C of interferon regulatory factor 2 (*IRF*-2) gene in freshwater mussel *Hyriopsis cumingii* were significantly related to *Aeromonas hydrophila* resistance (Wang et al., 2013). Because of the advantages with high genetic stability and high detection accuracy among many molecular markers, SNP marker has been widely used in the MAS of mollusks varieties with economic traits (Syvänen, 2001). In this study, we found that two SNP loci (c.323T > C and c.620C > T) showed a significant association with the ammonia tolerance of razor clams. Given that the genetic selection program with the increasing ammonia tolerance in *S. constricta* is still in the infancy, our findings suggest that the SNP c.323T > C and c.620C > T of *Sc-GDH* gene could be served as a potential genetic marker for MAS to increase survival rate and clam production.

As we all know, GDH plays a vital role in the metabolic pathway of ammonia nitrogen detoxification (Spanaki and Plaitakis, 2012). In this study, 26 SNPs were found in the CDS region of *Sc-GDH* (1 per 61 bp) in ZJ population, whereas 22 SNPs (1 per 72 bp) were observed in FJ population. The difference in numbers of SNP loci between two populations may be due to the genetic divergence or deviation of sequencing. The screenings of SNP in bivalves indicated that there was a high frequency of SNPs (1 per 40 bp) and insertion/deletion (1 per 33 bp) polymorphisms in ESTs (Saavedra and Bachère, 2006). Several studies suggested that average density of SNPs was estimated to be 1 per 60 bp in coding regions and 1 per 40 bp in noncoding regions in *Crassostrea gigas* (Sauvage et al., 2007). Identical conclusions were obtained in this study, reflecting that bivalves have abundant genetic variation. Moreover, the frequency of C–T was much higher than A–G one, which can be explained by that C in C&G sequence spontaneously exchanging to T via methylation (Yebra and Bhagwat, 1995; Yoon et al., 2001). Furthermore, the preference for tRNA binding to degenerate cordons in gene translation is not random; synonymous mutations may alter or reduce gene translation efficiency (Kimchi-Sarfaty et al., 2007). It has been shown that the synonymous mutations can be used to encode additional information to affect the speed or accuracy of mRNA translation, mRNA folding, mRNA splicing, and protein folding through translation pausing, thus changing its function (Supek et al., 2014; Zwart et al., 2018). In this regard, the two synonymous mutations with significant trait differences found in this study suggest their promising applications in the selective breeding of new varieties.

Immunohistochemistry can visualize target protein to achieve histocellular localization, which has become the most effective method for protein expression localization (Lugos et al., 2020). As we all know, the hepatopancreas tissue is an essentially digestive and detoxification organ (Wu et al., 2013). Under a high concentration of ammonia situation, edema, serious vacuolization, and local necrosis occur in liver, which in turn affect its detoxification function and even cause the death of fish (Hargreaves and Kucuk, 2001; Mishra and Mohanty, 2008). Similarly, in this study, Sc-GDH protein distributions altered after ammonia stress, which were mostly expressed in the cytoplasm endothelial cells of hepatocytes. Identical conclusions were found in the livers of chicken and mouse (Akutsu and Miyazaki, 2002; Vázquez-Martínez et al., 2017). Collectively, these findings indicate that Sc-GDH in the hepatopancreas of razor clam plays a key role in response to external ammonia entering the bodies.

RNA interference has evolved into a powerful tool for probing the functions of genes (Hannon, 2002), which has successfully led to the silence of homologous genes (Fire et al., 1998). Studies have shown that RNAi inhibition of *PmTNFR1* and *PmTNFR5* downregulated the downstream genes, which suggested that they mediated the *NF-κB* signaling pathway and were closely related to immune defense in the pearl oyster (Wu et al., 2020). Similarly, the silencing experiment of *LvGrx* 2 expression indicated that it was involved in the regulation of oxidative defense and antioxidant system in shrimp under ammonia stress (Zheng et al., 2019). In our previous study, *Sc-GDH* gene expression in hepatopancreas after exposure to ammonia stress (180 mg/L) was significantly higher than that of the controls (Zhang et al., 2020). In this study, we successfully silenced the expression of *Sc-GDH* by injecting siRNA, with 71.41% interference efficiency in AG at 48 h. Therefore, we propose to evaluate the long-term interference by injecting siRNA-*GDH* in the razor clam every 48 h to explore the downstream genes related to detoxification metabolism of ammonia stress in the future.

In conclusion, two SNPs in the CDS region of *Sc-GDH* gene were significantly associated with ammonia tolerance in two geographical populations, which could serve as candidate markers for MAS selection of ammonia-tolerant clams. In addition, the main secretion location of Sc-GDH was observed in the columnar cells of gill filaments and the endothelial cells of hepatocytes. Finally, we found that 48 h was the best effect duration on inhibiting *Sc-GDH* gene expression. These findings would contribute to clarify the role of *Sc-GDH* in ammonia tolerance and provide a foundational knowledge on the detoxification metabolism of razor clams under ammonia stress.

## Data Availability Statement

The datasets presented in this study can be found in online repositories. The names of the repository/repositories and accession number(s) can be found in the article/Supplementary Material.

## Ethics Statement

The animal study was reviewed and approved by Institutional Animal Care and Use Committee (IACUC) of Zhejiang Wanli University, China.

## Author Contributions

ZL, YD, and CS designed the study. GS performed the molecular analyses under the support of HY. Data analysis of the results was done by GS, YD, and CS. GS wrote the manuscript and all co-authors participated in the revisions of the manuscript. All authors contributed to the article and approved the submitted version.

## Conflict of Interest

The authors declare that the research was conducted in the absence of any commercial or financial relationships that could be construed as a potential conflict of interest.

## Footnotes

1 http://analysis.bio-x.cn/myAnalysis.php
