# Supplementary material for: Vital Role of Glutamate Dehydrogenase Gene in Ammonia Detoxification and the Association Between its SNPs and Ammonia Tolerance in Sinonovacula constricta
Source: Front Physiol. 2021 May 5;12:664804. doi: 10.3389/fphys.2021.664804 (PMC8131826; doi:10.3389/fphys.2021.664804)
Supplement: Supplementary file 1 [file Data_Sheet_1.docx]

Supplementary Material

# Supplementary Tables

**Table S1** Genotype, gene frequency and variation type of SNPs locus in *Sc-GDH*

| Locus/ Variation type | Genotype | ZJ | | | | FJ | | |
| --- | --- | --- | --- | --- | --- | --- | --- | --- |
|  |  | TG/CG | | χ2/*P* value | | TG/CG | | χ2/*P* value |
|  |  | Number | Genotype frequency |  |  | Number | Genotype frequency |  |
| c.269C > T/ Transition | CC | 51/39 | 38.64/32.23 | | 2.833/0.243 | 76/45 | 79.16/45 | 26.993/0.000 |
|  | CT | 56/56 | 42.42/46.28 | |  | 17/38 | 17.71/38 |  |
|  | TT | 25/26 | 18.94/21.49 | |  | 3/17 | 3.13/17 |  |
| **c.323T > C**/ Transition | TT | 46/38 | 34.85/31.41 | | **22.965/0.000** | 74/39 | 77.08/39 | **30.849/0.000** |
|  | TC | 57/25 | 43.18/20.66 | |  | 18/44 | 18.75/44 |  |
|  | CC | 29/58 | 21.97/47.93 | |  | 4/17 | 4.17/17 |  |
| c.401G > A/ Transition | GG | 71/71 | 53.79/58.68 | | 3.65/0.161 | 89/60 | 92.71/60 | 31.196/0.000 |
|  | GA | 54/38 | 40.91/31.41 | |  | 6/34 | 6.25/34 |  |
|  | AA | 7/12 | 5.3/9.91 | |  | 1/6 | 1.04/6 |  |
| c.611C > T/ Transition | CC | 94/79 | 71.21/65.29 | | 1.284/0.526 | 64/70 | 66.67/70 | 0.680/0.712 |
|  | CT | 34/36 | 25.76/29.75 | |  | 31/28 | 32.29/28 |  |
|  | TT | 4/6 | 3.03/4.96 | |  | 1/2 | 1.04/2 |  |
| **c.620C > T**/ Transition | CC | 53/27 | 40.15/22.32 | | **11.983/0.003** | 20/24 | 20.83/24 | **6.244/0.044** |
|  | CT | 57/57 | 43.18/47.1 | |  | 30/45 | 31.25/45 |  |
|  | TT | 22/37 | 16.67/30.58 | |  | 46/31 | 47.92/31 |  |
| c.677T > C/ Transition | TT | 39/18 | 29.54/14.88 | | 8.114/0.017 | 12/12 | 12.5/12 | 0.096/0.953 |
|  | TC | 55/58 | 41.67/47.93 | |  | 41/41 | 42.71/41 |  |
|  | CC | 38/45 | 28.79/37.19 | |  | 43/47 | 44.79/47 |  |
| c.749C > T/ Transition | CC | 60/54 | 45.45/44.63 | | 0.019/0.991 | 37/41 | 38.54/41 | 0.302/0.86 |
|  | CT | 55/51 | 41.67/42.15 | |  | 43/45 | 44.79/45 |  |
|  | TT | 17/16 | 12.88/13.22 | |  | 16/14 | 16.67/14 |  |
| c.752T > C/ Transition | TT | 97/77 | 73.49/63.64 | | 4.82/0.09 | 51/67 | 53.13/67 | 4.595/0.100 |
|  | TC | 28/29 | 21.21/23.97 | |  | 35/23 | 36.45/23 |  |
|  | CC | 7/15 | 5.3/12.39 | |  | 10/10 | 10.42/10 |  |
| c.839T > C/ Transition | TT | 118/111 | 89.39/91.73 | | 0.467/0.792 | 96/100 | - | - |
|  | TC | 13/9 | 9.85/10.74 | |  | 0/0 | - |  |
|  | CC | 1/1 | 0.76/0.83 | |  | 0/0 | - |  |
| c.848T > C/ Transition | TT | 29/21 | 21.97/17.36 | | 0.852/0.653 | 7/15 | 7.29/15 | 5.6228/0.06 |
|  | TC | 28/27 | 21.21/22.31 | |  | 40/28 | 41.67/28 |  |
|  | CC | 75/73 | 56.82/60.33 | |  | 49/57 | 41.04/57 |  |
| c.872C > T/ Transition | CC | 106/76 | 80.3/62.81 | | 10.125/0.006 | 74/70 | 77.08/70 | 4.032/0.133 |
|  | CT | 23/42 | 17.43/34.71 | |  | 19/20 | 19.79/20 |  |
|  | TT | 3/3 | 2.27/2.48 | |  | 3/10 | 3.13/10 |  |
| c.893G > A/ Transition | GG | 88/93 | 66.67/76.86 | | 3.577/0.167 | 96/100 | - | - |
|  | GA | 41/25 | 31.06/20.66 | |  | 0/0 | - |  |
|  | AA | 3/3 | 2.27/2.48 | |  | 0/0 | - |  |
| c.944C > T/ Transition | CC | 97/89 | 73.49/73.55 | | 0.968/0.616 | 62/69 | 64.58/69 | 0.564/0.754 |
|  | CT | 20/22 | 15.15/18.18 | |  | 25/24 | 26.04/24 |  |
|  | TT | 15/10 | 11.36/8.27 | |  | 9/7 | 9.38/7 |  |
| c.1001C > T/ Transition | CC | 47/38 | 35.61/31.41 | | 4.054/0.132 | 38/39 | 39.58/39 | 0.481/0.786 |
|  | CT | 25/36 | 18.94/29.75 | |  | 35/33 | 36.46/33 |  |
|  | TT | 60/47 | 45.45/38.84 | |  | 23/28 | 23.96/28 |  |
| c.1052C > T/ Transition | CC | 88/82 | 66.67/67.77 | | 0.351/0.839 | 74/66 | 77.08/66 | 3.129/0.209 |
|  | CT | 41/35 | 31.06/28.93 | |  | 19/28 | 19.79/28 |  |
|  | TT | 3/4 | 2.27/3.3 | |  | 3/6 | 3.13/6 |  |
| c.1106T > C/ Transition | TT | 100/82 | 75.76/67.77 | | 2.118/0.347 | 64/64 | 66.67/64 | 2.294/0.318 |
|  | TC | 28/33 | 21.21/27.27 | |  | 29/28 | 30.2/28 |  |
|  | CC | 4/6 | 3.03/4.96 | |  | 3/8 | 3.13/8 |  |
| c.1148T > C/ Transition | TT | 40/32 | 30.3/26.44 | | 0.477/0.788 | 35/32 | 36.46/32 | 0.479/0.787 |
|  | TC | 55/35 | 41.67/28.93 | |  | 42/48 | 43.75/48 |  |
|  | CC | 37/54 | 28.03/44.63 | |  | 19/20 | 19.79/20 |  |
| c.1169A > G/ Transition | AA | 93/84 | 70.46/69.42 | | 1.026/0.599 | 74/74 | 77.08/74 | 0.113/0.737 |
|  | AG | 36/36 | 27.27/29.75 | |  | 22/26 | 22.92/26 |  |
|  | GG | 3/1 | 2.27/0.83 | |  | 0/0 | - |  |
| c.1181T > G/ Transversion | TT | 73/40 | 55.3/33.06 | | 12.843/0.002 | 27/31 | 28.13/31 | 0.457/0.796 |
|  | TG | 37/49 | 28.03/40.5 | |  | 39/36 | 40.62/36 |  |
|  | GG | 22/32 | 16.67/26.44 | |  | 30/33 | 31.25/33 |  |
| c.1193C > T/ Transition | CC | 94/73 | 71.21/60.33 | | 4.24/0.12 | 54/56 | 56.25/56 | 0.91/0.637 |
|  | CT | 31/35 | 23.49/28.93 | |  | 35/33 | 36.46/33 |  |
|  | TT | 7/13 | 5.3/10.74 | |  | 7/11 | 7.29/11 |  |
| c.1229G > A/ Transition | GG | 94/81 | 71.21/66.94 | | 0.813/0.666 | 76/67 | 79.17/67 | 3.084/0.079 |
|  | GA | 35/38 | 26.52/31.41 | |  | 20/33 | 20.83/33 |  |
|  | AA | 3/2 | 2.27/1.65 | |  | 0/0 | - |  |
| c.1247C > T/ Transition | CC | 35/13 | 26.52/10.74 | | 10.582/0.005 | 16/11 | 16.67/11 | 1.337/0.512 |
|  | CT | 71/29 | 53.79/23.97 | |  | 32/35 | 33.33/35 |  |
|  | TT | 26/79 | 19.69/65.29 | |  | 48/54 | 50/54 |  |
| c.1301C > T/ Transition | CC | 96/80 | 72.73/66.11 | | 2.697/0.26 | 76/69 | 79.17/69 | 2.218/0.145 |
|  | CT | 33/40 | 25/33.06 | |  | 20/31 | 20.83/31 |  |
|  | TT | 3/1 | 2.27/0.83 | |  | 0/0 | - |  |
| c.1373A > C/ Transversion | AA | 68/31 | 51.51/25.62 | | 20.448/0.000 | 44/34 | 45.83/34 | 4.573/0.102 |
|  | AC | 46/54 | 34.85/44.63 | |  | 36/38 | 37.5/38 |  |
|  | CC | 18/36 | 13.64/29.75 | |  | 16/28 | 16.67/28 |  |
| c.1514G > A/ Transition | GG | 30/37 | 22.73/30.58 | | 3.547/0.17 | 96/100 | - | - |
|  | GA | 72/66 | 54.54/54.54 | |  | 0/0 | - |  |
|  | AA | 30/18 | 22.73/14.88 | |  | 0/0 | - |  |
| c.1598T > A/ Transversion | TT | 66/62 | 50/51.24 | | 2.563/0.278 | 96/100 | - | - |
|  | TA | 28/17 | 21.21/14.05 | |  | 0/0 | - |  |
|  | AA | 38/42 | 28.79/34.71 | |  | 0/0 | - |  |

Notes: The loci in bold black were those showed significant differences between the two populations.

**Table S2** Population genetic parameters of variable loci in *Sc-GDH*

| Population | Locus | TG | | | | CG | | | |
| --- | --- | --- | --- | --- | --- | --- | --- | --- | --- |
|  |  | *Ho* | *He* | *Ne* | *PIC* | *Ho* | *He* | *Ne* | *PIC* |
| ZJ | c.269C > T | 0.424 | 0.482 | 0.481 | 0.365 | 0.463 | 0.496 | 0.494 | 0.373 |
|  | c.323T > C | 0.432 | 0.494 | 0.492 | 0.371 | 0.207 | 0.488 | 0.486 | 0.369 |
|  | c.401G > A | 0.409 | 0.384 | 0.383 | 0.311 | 0.314 | 0.383 | 0.381 | 0.311 |
|  | c.611C > T | 0.258 | 0.269 | 0.268 | 0.233 | 0.298 | 0.319 | 0.318 | 0.269 |
|  | c.620C > T | 0.432 | 0.474 | 0.472 | 0.360 | 0.471 | 0.499 | 0.497 | 0.373 |
|  | c.677T > C | 0.417 | 0.502 | 0.500 | 0.375 | 0.479 | 0.477 | 0.475 | 0.363 |
|  | c.749T > C | 0.417 | 0.449 | 0.447 | 0.348 | 0.430 | 0.450 | 0.448 | 0.348 |
|  | c.752T > C | 0.212 | 0.269 | 0.268 | 0.233 | 0.240 | 0.370 | 0.369 | 0.298 |
|  | c.839T > C | 0.152 | 0.153 | 0.153 | 0.136 | 0.074 | 0.087 | 0.087 | 0.091 |
|  | c.848T > C | 0.212 | 0.441 | 0.439 | 0.344 | 0.223 | 0.409 | 0.408 | 0.327 |
|  | c.872C > T | 0.174 | 0.196 | 0.196 | 0.177 | 0.347 | 0.319 | 0.318 | 0.269 |
|  | c.893G > A | 0.311 | 0.294 | 0.293 | 0.252 | 0.207 | 0.224 | 0.223 | 0.201 |
|  | c.944C > T | 0.152 | 0.308 | 0.307 | 0.260 | 0.182 | 0.288 | 0.287 | 0.242 |
|  | c.1001C > T | 0.189 | 0.497 | 0.495 | 0.373 | 0.298 | 0.499 | 0.497 | 0.373 |
|  | c.1052C > T | 0.311 | 0.294 | 0.293 | 0.252 | 0.033 | 0.426 | 0.425 | 0.336 |
|  | c.1106T > C | 0.212 | 0.236 | 0.236 | 0.212 | 0.273 | 0.304 | 0.303 | 0.260 |
|  | c.1148T > C | 0.417 | 0.502 | 0.500 | 0.375 | 0.289 | 0.486 | 0.484 | 0.367 |
|  | c.1169A > G | 0.277 | 0.269 | 0.268 | 0.233 | 0.298 | 0.266 | 0.265 | 0.233 |
|  | c.1181T > G | 0.280 | 0.427 | 0.425 | 0.336 | 0.405 | 0.500 | 0.498 | 0.374 |
|  | c.1193C > T | 0.235 | 0.284 | 0.283 | 0.242 | 0.289 | 0.379 | 0.377 | 0.305 |
|  | c.1229G > A | 0.265 | 0.263 | 0.262 | 0.233 | 0.314 | 0.288 | 0.287 | 0.242 |
|  | c.1247C > T | 0.538 | 0.500 | 0.498 | 0.374 | 0.231 | 0.348 | 0.347 | 0.284 |
|  | c.1301C > T | 0.250 | 0.253 | 0.252 | 0.223 | 0.331 | 0.288 | 0.287 | 0.242 |
|  | c.1373A > C | 0.349 | 0.430 | 0.428 | 0.336 | 0.446 | 0.501 | 0.499 | 0.375 |
|  | c.1514G > A | 0.546 | 0.502 | 0.500 | 0.375 | 0.570 | 0.486 | 0.484 | 0.367 |
|  | c.1598T > A | 0.212 | 0.479 | 0.478 | 0.365 | 0.141 | 0.488 | 0.486 | 0.369 |
| FJ | c.269C > T | 0.788 | 0.211 | 1.267 | 0.189 | 0.537 | 0.461 | 1.855 | 0.355 |
|  | c.323T > C | 0.765 | 0.234 | 1.306 | 0.206 | 0.522 | 0.476 | 1.908 | 0.363 |
|  | c.401G > A | 0.920 | 0.080 | 1.087 | 0.074 | 0.644 | 0.354 | 1.549 | 0.292 |
|  | c.611C > T | 0.714 | 0.285 | 1.398 | 0.242 | 0.730 | 0.269 | 1.368 | 0.233 |
|  | c.620C > T | 0.534 | 0.463 | 1.863 | 0.356 | 0.450 | 0.498 | 1.990 | 0.374 |
|  | c.677T > C | 0.550 | 0.448 | 1.811 | 0.348 | 0.560 | 0.439 | 1.782 | 0.343 |
|  | c.749T > C | 0.521 | 0.476 | 1.909 | 0.363 | 0.534 | 0.464 | 1.864 | 0.356 |
|  | c.752T > C | 0.589 | 0.409 | 1.293 | 0.325 | 0.661 | 0.338 | 1.510 | 0.281 |
|  | c.848T > C | 0.594 | 0.404 | 1.692 | 0.322 | 0.586 | 0.412 | 1.700 | 0.327 |
|  | c872C > T | 0.772 | 0.227 | 1.679 | 0.201 | 0.678 | 0.320 | 1.471 | 0.269 |
|  | c.944C > T | 0.651 | 0.348 | 1.533 | 0.284 | 0.691 | 0.308 | 1.445 | 0.261 |
|  | c.1001C > T | 0.510 | 0.488 | 1.952 | 0.369 | 0.504 | 0.494 | 1.976 | 0.372 |
|  | c.1052C > T | 0.772 | 0.227 | 1.293 | 0.201 | 0.678 | 0.320 | 1.471 | 0.269 |
|  | c.1106T > C | 0.700 | 0.298 | 1.425 | 0.252 | 0.655 | 0.343 | 1.523 | 0.284 |
|  | c.1148T > C | 0.511 | 0.486 | 1.946 | 0.369 | 0.505 | 0.493 | 1.972 | 0.371 |
|  | c.1169A > G | 0.796 | 0.203 | 1.255 | 0.183 | 0.773 | 0.226 | 1.292 | 0.201 |
|  | c.1181T > G | 0.505 | 0.492 | 1.969 | 0.371 | 0.498 | 0.450 | 1.999 | 0.375 |
|  | c.1193C > T | 0.618 | 0.380 | 1.613 | 0.308 | 0.599 | 0.399 | 1.663 | 0.319 |
|  | c.1229G > A | 0.812 | 0.187 | 1.230 | 0.170 | 0.723 | 0.276 | 1.380 | 0.238 |
|  | c.1247C > T | 0.553 | 0.444 | 1.800 | 0.344 | 0.590 | 0.408 | 1.688 | 0.325 |
|  | c.1301C > T | 0.812 | 0.187 | 1.230 | 0.170 | 0.737 | 0.262 | 1.355 | 0.228 |
|  | c.1373A > C | 0.540 | 0.458 | 1.843 | 0.353 | 0.499 | 0.498 | 1.993 | 0.374 |
